# Comparative analysis of tissue-specific anticancer peptide prediction models: ACP-Boost framework

**DOI:** 10.3389/fmolb.2026.1815309

**Published:** 2026-03-26

**Authors:** Ruizhe Kang, Weichen Yuan, Mingjun Tang, Hongguang Zhou

**Affiliations:** 1 Department of Oncology, Affiliated Hospital of Nanjing University of Chinese Medicine, Nanjing, China; 2 Jiangsu Collaborative Innovation Center of Traditional Chinese Medicine Prevention and Treatment of Tumor, The First Clinical College of Nanjing University of Chinese Medicine, Nanjing, China

**Keywords:** anticancer peptides, cancer therapeutics, dataset construction, dipeptide composition, feature encodings, machine learning, tissue-specific classification, XGBoost

## Abstract

Cancer remains a major global health burden, and conventional treatments such as surgery, radiotherapy, and chemotherapy are often limited by systemic toxicity, drug resistance, and high cost. Anticancer peptides (ACPs) have emerged as promising therapeutic candidates because of their selective activity against tumor cells; however, experimental identification of ACPs is labor-intensive and time-consuming, and peptide activity may vary across tissue contexts. Although many computational models have been developed for general ACP prediction, tissue-specific ACP classification remains insufficiently explored. In this study, we developed ACP-Boost, a tissue-aware machine learning framework for tissue-specific ACP classification across nine cancer-related tissues: blood, brain, breast, cervix, colon, liver, lung, prostate, and skin. Experimentally validated peptide records were integrated from CancerPPD2 and DCTPep, followed by preprocessing, redundancy control, and tissue annotation. Peptide sequences were encoded into 473-dimensional feature vectors comprising amino acid composition (AAC), dipeptide composition (DPC), physicochemical property composition (PCP), and pseudo-amino acid composition (PseAAC). To address class imbalance while preserving the original sequence distribution, we formulated the task as a one-versus-rest classification problem, applied group-aware train-test splitting based on peptide sequences, and emphasized evaluation metrics suitable for imbalanced data. We systematically compared five machine learning algorithms, including support vector machine, random forest, logistic regression, k-nearest neighbors, and XGBoost. Among them, XGBoost showed the most stable overall performance across tissues. The results indicate that peptide sequence-derived descriptors contain measurable tissue-associated signals, although predictive separability remains moderate for several cancer types. Feature importance analysis further suggested that both shared charge-related properties and tissue-dependent sequence descriptors contribute to model discrimination. Overall, this study provides a comparative computational framework for tissue-specific ACP classification and highlights the importance of incorporating biological heterogeneity into peptide prediction tasks.

## 1 Introduction

Cancer represents one of the most formidable public health challenges worldwide, with approximately 20 million new cases diagnosed and nearly 10 million deaths recorded globally in 2022 alone (Bray et al., 2024). Despite considerable advances in oncology, surgical resection, cytotoxic chemotherapy, and radiotherapy, the cancer treatments remain burdened by fundamental limitations that substantially compromise both treatment outcomes and patient quality of life (Liu et al., 2024). Chemotherapy and radiotherapy, while conferring therapeutic benefit against rapidly proliferating tumor cells, are associated with severe systemic toxicities including cardiotoxicity, neurotoxicity, and immunosuppression. Moreover, Tumor cells can acquire resistance by upregulating drug efflux transporters, modifying drug targets, enhancing DNA damage repair capacity, and enriching cancer stem-like cell subpopulations, collectively driving disease progression and relapse (Li et al., 2025; Meng et al., 2021). These therapeutic challenges, compounded by the substantial economic burden imposed on patients and healthcare systems, highlight the critical need to develop novel antitumor strategies with improved selectivity, reduced systemic toxicity, and diminished susceptibility to resistance.

Anticancer peptides (ACPs) are a class of short peptides typically comprising 5 to 50 amino acids that exhibit selective cytotoxicity toward cancer cells while exerting minimal toxicity against normal tissues, and have emerged as promising candidates for novel antitumor therapeutics (Chinnadurai et al., 2023). Their selectivity is rooted in fundamental biophysical distinctions between malignant and normal cell membranes: aberrant phosphatidylserine exposure on the outer leaflet of cancer cell membranes, reduced cholesterol content, and enhanced membrane fluidity collectively elevate surface negative charge density, thereby promoting preferential electrostatic interactions with cationic peptide residues (Riedl et al., 2011). Beyond direct membrane disruption through pore formation and osmotic lysis, ACPs can exert synergistic antitumor effects through multiple complementary mechanisms (Wang et al., 2022), including activation of the mitochondrial apoptotic pathway (Karami Fath et al., 2022), suppression of tumor neovascularization (Rosca et al., 2011), and modulation of antitumor immune responses (Zare-Zardini et al., 2024). This mechanistic diversity, combined with structural plasticity and amenability to rational sequence modification, confers upon ACPs an intrinsic advantage over conventional chemotherapeutics in circumventing resistance development.

Given that experimental identification and characterization of ACPs through molecular biology approaches demand considerable time and financial investment (Giuliani et al., 2019), computational prediction methods have emerged as efficient alternatives for prioritizing candidate peptides prior to experimental validation (Basith et al., 2020). Over the past decade, numerous machine learning models have been developed for ACPs prediction, with notable examples including MLACP (Manavalan et al., 2017) and its successor MLACP 2.0 (Thi Phan et al., 2022), which employed Support Vector Machines and Random Forest algorithms; ACP-DL (Yi et al., 2019), which leveraged Long Short-Term Memory networks; and AntiCP 2.0 (Agrawal et al., 2021), a comprehensive web server integrating multiple algorithmic approaches. These models have consistently reported high performance metrics, with accuracy values routinely exceeding 90%, suggesting that computational ACPs prediction has achieved considerable progress.

However, a major limitation of current computational ACP predictors is that they are largely tissue-agnostic, formulating the task as generic ACP identification rather than tissue-resolved prediction. This simplification is difficult to reconcile with contemporary cancer biology, which emphasizes tissue-of-origin effects and organ-specific tumour microenvironments as major determinants of tumour phenotype and therapeutic response (Hoadley et al., 2018; Liang et al., 2021). Different cancer types arise within distinct tissue microenvironments characterized by unique molecular profiles, extracellular matrix compositions, vascular architectures, immune infiltration patterns, and metabolic states (Junttila and de Sauvage, 2013), all of which collectively determine peptide bioavailability, cellular uptake, target accessibility, and therapeutic efficacy. The blood-brain barrier restricts peptide access to brain tumors through tight junction proteins and active efflux transporters (Oberoi et al., 2016). Liver tumors reside within metabolically hyperactive hepatic parenchyma characterized by extensive proteolytic activity (Mahmood and Pettinato, 2021), substantially compromising peptide pharmacokinetics. Hematological malignancies entirely lack solid tumor architecture, fundamentally altering the requirements for therapeutic targeting. Bovine lactoferricin B selectively inhibited gastric cancer cells with an IC50 of 64 μM, while demonstrating markedly variable potency across other cancer types (Pan et al., 2013; Arias et al., 2017). Neuropeptide Y promotes tumor cell proliferation in breast cancer and neuroblastoma, yet suppresses tumor growth in hepatocellular carcinoma, depending on tissue-specific receptor subtype expression patterns unique to each tumor context (Sigorski et al., 2025). These observations collectively underscore a critical conclusion: a peptide demonstrating potent activity against one cancer type may exhibit absent activity against another, rendering tissue-agnostic prediction models severely limited in clinical utility (Piktel et al., 2016; Tyagi et al., 2015).

In this study, we developed a systematic evaluation framework for tissue-specific anticancer peptide prediction based on multiple machine learning algorithms. Specifically, our work has three main objectives. First, we establish benchmark performance baselines for tissue-specific ACP classification across nine cancer-related tissues. Second, we compare the behavior of different machine learning models under tissue-specific settings in order to identify algorithms with the most stable and effective predictive performance. Third, we investigate biologically interpretable sequence patterns through feature importance analysis and cross-tissue comparison, with the aim of linking predictive signals to potential tissue-associated mechanisms. Taken together, this study provides a standardized computational framework for tissue-specific ACP prediction and may support the rational screening and optimization of peptide candidates for targeted anticancer applications.

## 2 Materials and methods

### 2.1 Data collection and curation

Experimentally validated anticancer peptide sequences were collected from two publicly available databases: CancerPPD2 (Chauhan et al., 2025) (http://webs.iiitd.edu.in/raghava/cancerppd2/) and DCTPep (Sun et al., 2024) (https://www.dctpep.com/). Because the two resources contain partially overlapping entries, duplicate sequences were identified and merged while retaining the most complete annotation available.

The combined dataset was filtered to focus on nine major cancer tissue types with sufficient representation for robust modeling: Blood, Brain, Breast, Cervix, Colon, Liver, Lung, Prostate, and Skin. Tissue labels were assigned using keyword-based matching of cancer type, disease annotation, and cell line information. Entries with ambiguous or conflicting annotations were excluded.

All peptide sequences were converted to uppercase and restricted to the 20 standard amino acids. Sequences shorter than 10 residues or longer than 100 residues were removed. Quantitative activity information, including IC50 values where available, was extracted from the original annotations and standardized to μM. Entries lacking essential sequence or tissue information were discarded during preprocessing. For tissue-specific prediction, we formulated the task as a one-versus-rest classification problem. For each tissue, peptides annotated to that tissue were treated as positive samples, whereas peptides associated with the remaining tissues were treated as negative samples. This formulation directly reflects the goal of tissue-specific ACP classification and avoids the instability caused by extremely sparse within-tissue inactive annotations.

### 2.2 Feature engineering

Raw peptide sequences, represented as variable-length amino acid strings, cannot be directly used as input for conventional machine learning algorithms. Therefore, each peptide was encoded into a fixed-length 473-dimensional numerical feature vector capturing sequence composition, physicochemical characteristics, and short-range sequence-order information.

Feature extraction was implemented in a custom Python pipeline using four descriptor families: amino acid composition (AAC, 20 features), dipeptide composition (DPC, 400 features), physicochemical property composition (PCP, 30 features), and pseudo-amino acid composition-like descriptors (PAAC, 23 features). In this implementation, PCP features consisted of residue group compositions and additional simple physicochemical ratio features, whereas PAAC features were computed using three standardized amino acid property scales (hydrophobicity, hydrophilicity, and residue mass) with λ = 3 and w = 0.05, yielding a total of 473 features.

#### 2.2.1 Amino acid composition (AAC) – 20 features

AAC (Gasteiger et al., 2005) captures the global frequency of each of the 20 standard amino acids in a peptide sequence, providing fundamental compositional information. For a peptide sequence S of length N containing $R_{i}$ occurrences of amino acid type i :

$$AAC_{i}=\frac{R_{i}}{N}$$

where $R_{i}$ denotes the number of residues of amino acid type i ,

$$i\in \left\{A,C,D,E,F,G,H,I,K,L,M,N,P,Q,R,S,T,V,W,Y\right\}$$

N is the sequence length, and AAC values range from 0 to 1, with higher values indicating enrichment of specific amino acids. This simple yet effective representation has been widely used in protein function prediction and has proven particularly informative for anticancer peptides, where amino acid composition (especially cationic and hydrophobic residues) correlates with membrane-lytic activity.

#### 2.2.2 Dipeptide composition (DPC) – 400 features

DPC extends AAC by encoding the frequencies of all possible adjacent amino acid pairs, thereby incorporating short-range sequence-order information. For each dipeptide type ij ,DPC is defined as:

$$DPC_{ij}=\frac{N_{ij}}{N-1}$$

where $N_{ij}$ represents the count of dipeptide ij in the sequence, and N denotes the sequence length,and

$$i,j\in \left\{A,C,D,E,F,G,H,I,K,L,M,N,P,Q,R,S,T,V,W,Y\right\}$$

This generates 20×20=400 features representing all possible dipeptide combinations (e.g., AA, AC, AD, …, YW, YY). DPC has proven valuable for peptide activity prediction because specific dipeptide motifs (e.g., KK, RR for cationic regions; LL, II for hydrophobic domains) are often enriched in bioactive peptides and contribute to functional activity through conformational preferences and interaction propensities.

#### 2.2.3 Physicochemical property composition (PCP) – 30 features

PCP features were computed as a combination of residue-group composition descriptors and additional simple physicochemical ratio/statistic descriptors, following established sequence-derived physicochemical descriptor frameworks based on amino acid property groupings (Dubchak et al., 1995; Pande et al., 2023). Specifically, in this study, we defined 20 residue-group composition features based on predefined amino acid groupings, including Positive, Negative, Polar, NonPolar, Hydrophobic, Hydrophilic, Aromatic, Aliphatic, Sulfur, Tiny, Small, Large, Charged, Neutral, Flexible, Rigid, HelixFormer, SheetFormer, TurnFormer, and Intermediate groups. For each group i , composition was calculated as:

$$PCP_{i}=\frac{P_{i}}{N}$$

where $P_{i}$ is the number of residues belonging to group i and *N* is the peptide length. In addition, 10 extra descriptors were included: hydrophobic ratio, polar ratio, charged ratio, net charge, average hydrophobicity, aromatic ratio, aliphatic ratio, helix-forming residue ratio, sheet-forming residue ratio, and turn-forming residue ratio. Together, these 30 PCP features provide a higher-level representation of peptide physicochemical characteristics.

#### 2.2.4 Pseudo-amino acid composition (PseAAC) – 23 features

PseAAC (Chou, 2001) extends traditional AAC by incorporating sequence-order information through correlation factors that capture physicochemical property relationships between amino acids at different positions along the sequence. This representation preserves both composition and sequential effects.

For sequence-order correlation with lag λ , PseAAC computes correlation between amino acids separated by λ positions based on their hydrophobicity, hydrophilicity, and side-chain mass properties. The standard normalization converts raw property values $H^{\circ }\left(i\right)$ to normalized values $H\left(i\right)$ :

$$H\left(i\right)=\frac{H^{\circ }\left(i\right)-\text{mean}\left(H^{\circ }\right)}{\text{std}\left(H^{\circ }\right)}$$

where mean and standard deviation are computed across all 20 amino acids. For this study, λ = 3 was selected, generating 20 normalized amino acid frequencies plus three sequence-order correlation factors, totaling 23 features. The λ = 3 configuration captures local sequence patterns up to 3-residue gaps while avoiding excessive feature dimensionality. PseAAC has demonstrated superior performance compared to simple AAC in numerous peptide activity prediction tasks by encoding both what amino acids are present and how they are arranged. All feature calculations were performed using Pfeature’s standardized mplementations with default parameters. The resulting 473-dimensional feature vectors (20 AAC +400 DPC +30 PCP +23 PseAAC) provide comprehensive representation of peptide sequence characteristics relevant to anticancer activity while maintaining computational tractability for machine learning.

### 2.3 Data splitting and imbalance considerations

For tissue-specific prediction, each task was formulated as a one-versus-rest classification problem (Rifkin and Klautau, 2004), in which peptides annotated to the target tissue were treated as positive samples and peptides associated with the remaining tissues were treated as the reference class. Synthetic oversampling methods such as SMOTE were not applied, because artificially generated peptide samples may distort the original sequence-feature distribution and reduce biological interpretability in a relatively limited tissue-stratified dataset (Fernández et al., 2018). Accordingly, class imbalance was not addressed through synthetic resampling, but instead considered through task formulation (Hou et al., 2022), sequence-aware data partitioning, algorithm-level weighting, and the use of evaluation metrics suitable for skewed class distributions.

To minimize information leakage, data partitioning was performed using group-aware splitting based on peptide sequences, such that all entries corresponding to the same peptide sequence were assigned to a single partition. This strategy prevented identical sequences from appearing in both the training and test sets. Model performance was evaluated using multiple complementary metrics, with Matthews correlation coefficient (MCC) and area under the precision-recall curve (AUC-PR) treated as key metrics because they are more informative than accuracy alone in imbalanced one-versus-rest classification settings (Saito and Rehmsmeier, 2015; Chicco and Jurman, 2020).

### 2.4 Machine learning algorithms

Five machine learning algorithms representing diverse learning paradigms were evaluated for tissue-specific ACPs prediction, selected based on their established effectiveness in biological sequence classification and complementary modeling characteristics.

#### 2.4.1 Support vector machine (SVM)

SVM (Cortes and Vapnik, 1995) with linear kernel was employed to identify the optimal separating hyperplane in the 473-dimensional feature space. Linear SVM is particularly well-suited for high-dimensional data (features >> samples) and often achieves competitive performance through effective margin maximization. The algorithm seeks to maximize the margin between classes while minimizing classification errors through the optimization problem:

$$\min_{w,b,\xi }\;\frac{1}{2}{\left\|w\right\|}^{2}+C\sum_{i=1}^{n}\xi_{i}$$

subject to $y_{i}\left(w\cdot x_{i}+b\right)\geq 1-\xi_{i},i=1,\ldots ,n$ ,where w is the hyperplane normal vector, C is the regularization parameter, and are slack variables permitting soft-margin violations. The model was implemented using scikit-learn SVC with kernel = 'linear’, class_weight = 'balanced’, and probability = True to enable probability estimation via Platt scaling.

#### 2.4.2 Random forest (RF)

Random Forest (Breiman, 2001) is an ensemble learning method that constructs multiple decision trees on bootstrap samples of training data with random feature subsets at each split, then aggregates predictions via majority voting. This approach reduces overfitting inherent in single decision trees while capturing complex nonlinear decision boundaries.

Each tree is grown by recursively partitioning the feature space to maximize information gain (or minimize Gini impurity), considering only a random subset of $\sqrt{473}$ ≈ 22 features at each split. The final prediction aggregates across all trees, providing built-in ensemble uncertainty quantification.

Implementation: scikit-learn RandomForestClassifier with class_weight = 'balanced’, oob_score = True (enabling out-of-bag error estimation as an internal validation metric), n_jobs = −1 (parallel processing across all CPU cores), and random_state = 42 (reproducibility).

#### 2.4.3 Logistic regression (LR)

Logistic Regression, despite being a linear model, often performs competitively on high-dimensional data due to regularization and probabilistic interpretation. The algorithm models the log-odds of class membership as a linear function:

$$\log\frac{P\left(y=1|x\right)}{P\left(y=0|x\right)}=w\cdot x+b$$

Regularization using either L1 or L2 penalties was applied to prevent overfitting by constraining coefficient magnitudes, with L1 regularization additionally encouraging sparsity and implicit feature selection.

The model was implemented using LogisticRegression from scikit-learn with class_weight = 'balanced’, max_iter = 1,000 to ensure convergence, and solver selection optimized during hyperparameter tuning (liblinear for L1 penalty and lbfgs for L2 penalty).

#### 2.4.4 K-nearest neighbors (KNN)

KNN classifies a query sample by majority vote among its k nearest neighbors in feature space, making no parametric assumptions about the underlying data distribution. The choice of k, distance metric (Euclidean or Manhattan), and voting scheme (uniform or distance-weighted) were optimized via hyperparameter tuning. The model was implemented using scikit-learn KNeighborsClassifier.

#### 2.4.5 Extreme gradient boosting (XGBoost)

XGBoost (Chen and Guestrin, 2016) is a gradient boosting framework that iteratively constructs an ensemble of decision trees, where each subsequent tree corrects errors made by previous trees. The algorithm minimizes a regularized objective function:

$$Obj=\sum_{i=1}^{n}L\left(y_{i},{\hat{y}}_{i}\right)+\sum_{k=1}^{K}\Omega \left(f_{k}\right)$$

where L is a differentiable loss function and $\Omega \left(f_{k}\right)$ penalizes tree complexity to prevent overfitting. The model was implemented using XGBoost (version 1.5.0) with objective = 'binary:logistic’, eval_metric = 'logloss’, and tree_method = 'hist’. To account for residual class imbalance in tissue-specific subsets without introducing synthetic samples, scale_pos_weight was tuned within the training process when appropriate.

### 2.5 Hyperparameter optimization and model training

For each tissue-specific task, data were partitioned into training (80%) and test (20%) sets using group-aware splitting based on peptide sequences to avoid information leakage (Hou et al., 2022). For models sensitive to feature scale, standardization was performed using parameters estimated from the training set only and then applied to the test set. Tree-based models were trained on the original feature values. Hyperparameter selection was conducted exclusively within the training data (Bergstra and Bengio, 2012). In particular, XGBoost hyperparameters were optimized by randomized search with group-based cross-validation, using AUC-PR as the model-selection criterion. Class imbalance was handled through algorithm-level weighting strategies rather than synthetic resampling. Final model performance was then evaluated on the held-out test set. The hyperparameter search space for all evaluated machine learning models is summarized in Table 1.

**TABLE 1:** Hyperparameter search space for machine learning models.

Model	Hyperparameter	Search values
SVM	C	0.001, 0.01, 0.1, 1, 10
	kernel	linear
Random forest	n_estimators	10, 50, 100, 200
	max_depth	2, 3, 5, 7, 10
	min_samples_leaf	5, 10, 20
Logistic regression	C	0.001, 0.01, 0.1, 1, 10
	penalty	l1, l2
	solver	liblinear, lbfgs, saga
KNN	n_neighbors	3, 5, 7, 9, 11
	weights	uniform, distance
	metric	manhattan, euclidean
XGBoost	learning_rate	0.01, 0.05, 0.1, 0.3
	max_depth	3, 5, 7
	n_estimators	50, 100, 200
	subsample	0.8, 1.0
	colsample_bytree	0.8, 1.0

### 2.6 Performance evaluation metrics

Model performance was evaluated using balanced accuracy (Brodersen et al., 2010), accuracy, precision, recall, F1-score, Matthews correlation coefficient (MCC), area under the ROC curve (AUC-ROC), and area under the precision-recall curve (AUC-PR) (Saito and Rehmsmeier, 2015). In the one-versus-rest setting, positive samples refer to peptides associated with the target tissue, whereas negative samples correspond to peptides from the remaining tissues. Balanced accuracy was included because it is less sensitive to majority-class dominance and better reflects classification performance under imbalanced conditions. It was calculated as the average of sensitivity and specificity.

The metrics were defined as follows:

$$Accuracy=\frac{TP+TN}{TP+TN+FP+FN}$$

$$Precision=\frac{TP}{TP+FP}$$

$$Recall=\frac{TP}{TP+FN}$$

$$F1=\frac{2\times Precision\times Recall}{Precision+Recall}$$

$$MCC=\frac{TP\times TN-FP\times FN}{\sqrt{\left(TP+FP\right)\left(TP+FN\right)\left(TN+FP\right)\left(TN+FN\right)}}$$

$$Balanced\;Accuracy=\frac{1}{2}\left(\frac{TP}{TP+FN}+\frac{TN}{TN+FP}\right)$$

AUC-ROC and AUC-PR were computed from predicted probabilities. Among these metrics, MCC was considered the primary evaluation metric because it provides a balanced assessment under class imbalance. AUC-PR was additionally emphasized during model optimization, as it is more informative than ROC-based measures in imbalanced one-versus-rest classification settings. All metrics were computed on held-out test sets using scikit-learn, and average values across tissues were used for overall model comparison.

### 2.7 Feature importance analysis

Feature importance analysis was performed for the tree-based models, Random Forest and XGBoost, to identify sequence descriptors contributing to tissue-specific prediction. Importance scores were obtained from the built-in feature importances attribute of each model (Huynh-Thu et al., 2012). For each tissue, the highest-ranked features were summarized according to descriptor category, including AAC, DPC, PCP, and PseAAC, to facilitate biological interpretation of tissue-associated sequence patterns.

### 2.8 High-confidence prediction identification

High-confidence predictions were explored descriptively for illustrative purposes, but were not used in the primary evaluation of model performance.

### 2.9 Statistical analysis

Descriptive statistics were used to summarize model performance across tissues. For each algorithm, average performance metrics were calculated across the nine one-versus-rest tasks, and cross-tissue variability was described using standard deviation. Algorithms were compared primarily based on average MCC, AUC-PR, and average rank across tissues.

### 2.10 Computational environment

All analyses were implemented in Python 3.9.7. Key software dependencies are summarized in Table 2. Computational experiments were executed on a high-performance computing cluster running Ubuntu 20.04 LTS, equipped with Intel Xeon E5-2680 v4 processors (2.40 GHz, 14 cores per node) and 32 GB RAM per node. Parallelizable operations utilized all available CPU cores. Total computational time for all experiments (9 tissues × 5 algorithms with hyperparameter optimization) was approximately 48 h.

**TABLE 2:** Software packages and versions used in this study.

Package	Version	Purpose
Python	3.9.7	Programming environment
scikit-learn	1.0.2	Machine learning algorithms, preprocessing, metrics
XGBoost	1.5.0	Gradient boosting implementation
Pfeature	1.0.2	Peptide feature extraction
pandas	1.3.4	Data manipulation
NumPy	1.21.2	Numerical computing
SciPy	1.7.1	Statistical analysis
matplotlib	3.4.3	Visualization

## 3 Results and discussions

### 3.1 Dataset characteristics and preprocessing

**FIGURE 1:**
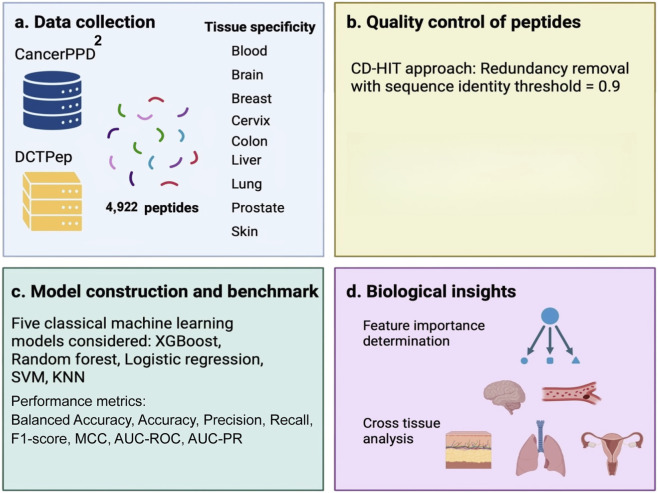
Schematic workflow of the ACP-Boost framework for tissue-specific anticancer peptide classification. The workflow comprises four main components: **(a)** data collection; **(b)** peptide quality control; **(c)** model construction and benchmarking; and **(d)** biological insights.

The overall ACP-Boost framework is illustrated in Figure 1. The workflow integrates data collection and curation from CancerPPD2 and DCTPep, redundancy removal, tissue-specific one-versus-rest (OVR) model construction, and biological interpretation through feature importance analysis.

**FIGURE 2:**
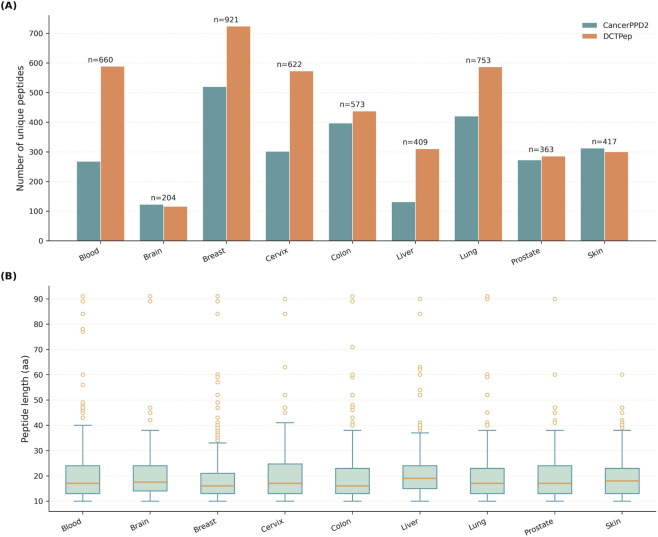
Dataset characteristics of the integrated peptide cohort across nine tissue-specific classes. **(A)** Distribution of unique peptides across tissues, stratified by data source (CancerPPD2 and DCTpep). Numbers above bars indicate total unique peptides in each tissue. **(B)** Distribution of peptide lengths across tissues.

The integrated dataset comprised 4,922 tissue-specific entries corresponding to 2,371 unique peptide sequences across the nine tissue categories. Each entry represents a unique sequence-tissue pair; therefore, the same peptide could contribute to more than one tissue-specific instance if activity annotations were available in multiple tissues. As shown in Figure 2A, the numbers of entries varied substantially across tissues. Breast and lung were the most highly represented categories, whereas brain was the smallest subset, with liver and prostate also containing relatively fewer entries. In addition, both CancerPPD2 and DCTPep contributed to all tissue categories, although their relative proportions differed across tissues. This uneven distribution indicates that the prediction tasks were intrinsically imbalanced across tissues, which is likely to affect model stability and may partly explain cross-tissue differences in predictive performance.

Peptide length distributions are shown in Figure 2B. Across all tissues, peptide lengths were concentrated within a broadly similar range, with median values clustered at approximately 15–20 amino acids and moderate interquartile variation. A limited number of longer outliers were also observed in several tissues, but no category displayed a pronounced systematic shift toward substantially shorter or longer sequences. This overall consistency suggests that the integrated dataset does not exhibit a strong tissue-specific length bias. Therefore, differences in model performance across tissues are more likely to reflect variation in sequence composition and physicochemical characteristics than trivial differences in peptide length alone.

### 3.2 Overall model performance

**FIGURE 3:**
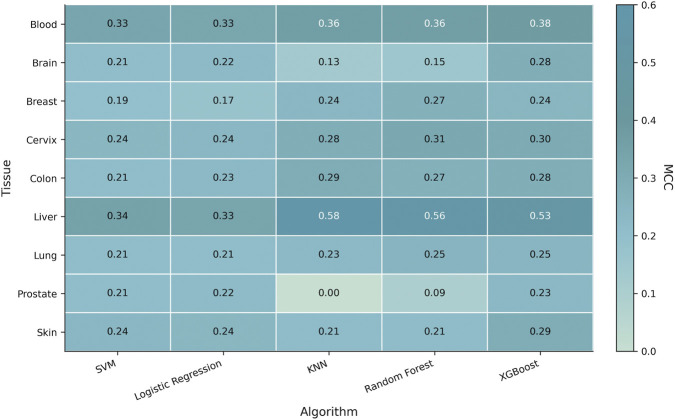
Tissue-specific benchmark performance of five classical machine learning algorithms across nine one-versus-rest classification tasks. Heatmap cells represent Matthews correlation coefficient (MCC) values for each algorithm–tissue combination. Higher values indicate better classification performance.

In tissue-specific ACP identification, we systematically evaluated five classical machine learning algorithms, namely, support vector machine (SVM), random forest (RF), logistic regression (LR), k-nearest neighbor (KNN), and extreme gradient boosting (XGBoost). Their overall performance across nine tissue-specific one-versus-rest classification tasks is presented in Figure 3 and Supplementary Table S1. Overall, substantial heterogeneity in predictive performance was observed across both algorithms and tissues, indicating that the difficulty of tissue-specific ACP identification is determined not only by the choice of model, but also by the biological context of the target tissue, sample size, and sequence diversity. Nevertheless, based on the mean Matthews correlation coefficient (MCC) across the nine tasks, XGBoost achieved the best overall performance and was therefore selected as the primary model for subsequent in-depth analysis and biological interpretation.

As shown in Figure 3, the superiority of XGBoost was not confined to a single tissue, but was reflected in its stronger overall robustness and generalization ability. XGBoost achieved the highest MCC in several tasks, including Blood, Brain, Prostate, and Skin, and was also tied for the best performance in Lung. Even in tissues where it did not rank first, its performance was generally close to that of the best-performing model. This suggests that, relative to the other algorithms, XGBoost was more capable of consistently capturing tissue-related signals embedded in ACP sequences across different tissue contexts, rather than excelling only in isolated data subsets. By contrast, although RF and KNN showed strong performance in certain tissues, such as Liver, Colon, or Cervix, their advantages appeared to be more task-specific and less consistent overall than those of XGBoost. SVM and LR, on the other hand, showed comparatively conservative performance, suggesting that linear or relatively simple decision boundaries may be insufficient to fully characterize the complex sequence patterns underlying tissue-specific ACPs.

From a biological perspective, these findings are plausible. AAC, DPC, PCP, and PseAAC descriptors together define a high-dimensional feature space that integrates compositional information, local sequence-order information, and physicochemical properties. Within such a space, nonlinear relationships and higher-order interactions among features are likely to exist. As a boosting-based ensemble tree model, XGBoost is better suited to modeling these complex patterns and therefore exhibits greater adaptability in tissue-specific prediction. In other words, tissue origin does appear to leave detectable signatures in ACP sequences, but these signatures are unlikely to be simply linearly separable; rather, they are more likely to arise from the joint effects of residue composition, local dipeptide patterns, and physicochemical properties. Under such circumstances, XGBoost is more capable than SVM, LR, or KNN of extracting discriminative combinatorial signals, which may partly explain its overall superior performance.

**FIGURE 4:**
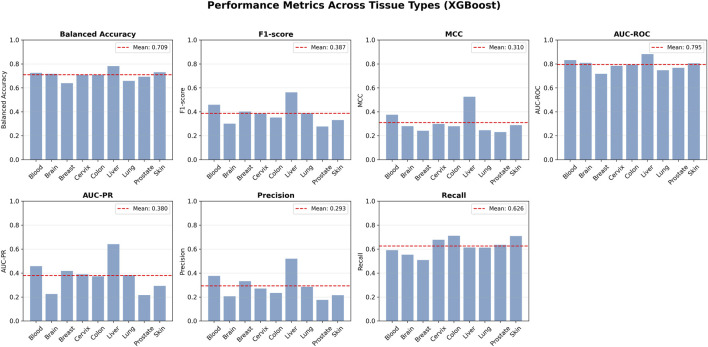
Tissue-wise held-out test performance of the XGBoost models across nine one-versus-rest classification tasks. Metrics shown include balanced accuracy, F1-score, MCC, AUC-ROC, AUC-PR, precision, and recall. Red dashed lines indicate the mean value of each metric across the nine tissue types.

To further assess the best-performing model at the tissue level, we examined the held-out test results of XGBoost across the nine one-versus-rest tasks in greater detail (Figure 4). Overall, XGBoost showed moderate but stable discriminative ability across tissues, with mean Balanced Accuracy, MCC, AUC-ROC, AUC-PR, and F1-score values of 0.709, 0.310, 0.795, 0.380, and 0.387, respectively. These results indicate that, although tissue-specific ACP classification remains a challenging task, sequence-derived descriptors nonetheless provide reproducible and informative tissue-associated signals. Of particular note, AUC-ROC remained relatively high and stable across most tissues, suggesting that the model performed reliably in terms of overall ranking ability. By contrast, MCC and AUC-PR varied more markedly between tissues, further reflecting genuine differences in classification difficulty under the inherently imbalanced one-versus-rest setting.

At the level of individual tissues, Liver showed the strongest performance across multiple metrics and appeared to be one of the most readily separable tissue categories. Blood and Skin also showed comparatively favorable predictive results. In contrast, Brain and Prostate were more difficult to classify, suggesting that ACPs associated with these tissues may overlap more extensively with those from other tissues in sequence-feature space, or that the learnable signal may be constrained by sample size, label heterogeneity, or sequence diversity. Importantly, such cross-tissue variation does not diminish the value of the model; rather, it underscores that tissue-specific ACPs do not represent a homogeneous category, and that different tissues may be associated with distinct degrees of sequence constraint and mechanistic specificity. Thus, the model is not merely performing classification, but also indirectly reflecting the extent to which ACP patterns associated with different tissues are separable.

In addition, Figure 4 shows that recall was generally higher than precision for XGBoost across most tissues. This pattern suggests that, under the current setting, the model tended to prioritize the identification of potential positive peptides rather than excluding candidate samples too conservatively. For the practical task of tissue-specific ACP screening, this characteristic should not necessarily be regarded as a weakness; on the contrary, it may be advantageous. In the candidate discovery stage, higher recall allows the model to retain as many potentially active peptides as possible, thereby reducing the risk of missing true positives. Subsequent experimental validation or more stringent downstream filtering can then be used to control the impact of false positives. In this sense, XGBoost appears to adopt a prediction strategy better suited to preliminary screening, namely, one that prioritizes sensitivity to positive samples while maintaining reasonable MCC and Balanced Accuracy (Hicks et al., 2022).

Taken together, these findings indicate that tissue-specific ACP classification is biologically meaningful, although it is not a task that can be cleanly separated in all cases. This suggests that distinct tissues are indeed associated with sequence-level signals that can be captured by peptide descriptors, although these signals remain only partially overlapping and incompletely separable. Among the five algorithms evaluated, XGBoost demonstrated the best overall performance, superior cross-tissue stability, and a prediction profile more suitable for candidate screening. Its selection as the core model for subsequent feature-importance analysis and tissue-level interpretation is therefore both justified and well supported. More broadly, the tissue-aware predictive framework built on XGBoost was able to capture sequence patterns associated with tissue context in a relatively stable manner, patterns that would likely be weakened or obscured in a tissue-agnostic modeling strategy.

### 3.3 Feature importance analysis

**FIGURE 5:**
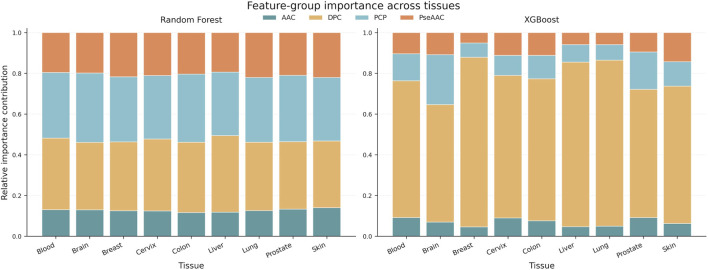
Feature-group importance across nine tissue-specific one-versus-rest ACP classification tasks. Stacked bars summarize the total contribution of AAC, DPC, PCP, PseAAC, and other descriptor groups in the Random Forest and XGBoost models across tissues. Feature importance was derived from the built-in importance scores of each model and aggregated by descriptor category for cross-tissue comparison.

To further elucidate the sequence basis underlying tissue-specific discrimination, we compared the feature-importance profiles of the Random Forest (RF) and XGBoost models, with particular emphasis on XGBoost, which achieved the best overall predictive performance (Figure 5; Supplementary Table S2). At the feature-group level, the two models exhibited clearly distinct attribution patterns. RF showed a relatively diffuse dependence on AAC, PCP, and PseAAC features, with a more balanced distribution of importance across feature types. By contrast, XGBoost displayed a much more concentrated reliance on DPC features. When the top 20 most important features from each of the nine tissues were pooled, 129 of the 180 features selected by XGBoost belonged to the DPC category, whereas PCP, AAC, and PseAAC accounted for only 22, 12, and 17 features, respectively. In contrast, among the 180 top-ranked features identified by RF, 103 were PCP features and 60 were PseAAC features, whereas only 2 DPC features appeared among the leading variables. These findings indicate that, although both models were capable of extracting tissue-related information from peptide sequences, they differed substantially in the way such information was utilized: RF relied more heavily on global physicochemical context and overall sequence-order trends, whereas XGBoost preferentially identified discriminative sequence patterns embedded in local dipeptide combinations.

This difference is not only methodologically relevant, but also informative with respect to how tissue-specific information may be encoded in ACP sequences. AAC, PCP, and PseAAC features largely represent summaries of overall composition, physicochemical profile, and low-order sequence-order information, whereas DPC directly preserves local pairing relationships between adjacent residues. The pronounced preference of XGBoost for DPC features therefore suggests that tissue-specific information may not be expressed primarily as simple shifts in the overall abundance of particular amino acids, but rather as differences in the local arrangement of residues. In other words, the key signal determining tissue assignment may lie less in which residues are present *per se* than in how they are locally organized along the sequence. This observation may also partly explain the overall advantage of XGBoost over the other models: in a high-dimensional feature space characterized by nonlinear relationships and higher-order interactions, XGBoost appears better able to extract stable discriminative signals from local, combinatorial sequence patterns.

Importantly, the strong preference of XGBoost for DPC features cannot be attributed solely to the larger number of DPC variables. Although the DPC feature set comprises 400 descriptors, far exceeding the dimensionality of AAC, PCP, and PseAAC, a similar pattern would be expected in RF if dimensionality alone were responsible for this bias. However, DPC features were almost entirely absent from the major feature set of RF, indicating that the observed difference more likely reflects a genuine preference of the models for different forms of information rather than a simple dimensionality effect. In this sense, XGBoost appears to preferentially exploit motif-like local signals, whereas RF favors the integration of smoother, cumulative physicochemical statistics. This contrast in attribution patterns suggests that the sequence signals underlying tissue-specific ACP discrimination may themselves be hierarchical in nature, with different models capturing different levels of that structure.

When examined at the tissue level, the feature-importance landscape of XGBoost did not conform to a single uniform pattern, but instead showed substantial inter-tissue variation. Broadly, three general patterns could be distinguished. The first may be described as a local motif-dominant pattern, most evident in Breast, Liver, and Lung. In these tissues, the top-ranked features were overwhelmingly dominated by DPC descriptors, suggesting that their separability depended to a considerable extent on local adjacent-residue patterns rather than primarily on global compositional shifts. The second may be described as a jointly driven pattern involving local motifs and global physicochemical features, as observed in Blood, Cervix, Colon, and Skin. Although DPC features still predominated in these tissues, AAC, PCP, or PseAAC descriptors also made non-negligible contributions, indicating that tissue-associated signals in these contexts were not determined exclusively by local motifs, but instead reflected a combination of short-range sequence-order information and broader physicochemical background. The third pattern may be characterized as integrated physicochemical constraint-dominant, with Brain and Prostate being the most representative examples. In Brain, the highest-ranked feature was PCP_Intermediate, accompanied by multiple PCP descriptors such as PCP_Positive, PCP_Aliphatic_Ratio, and PCP_TurnFormer; in Prostate, PCP_Tiny ranked first, together with AAC_H, AAC_R, PAAC_H, and several descriptors related to turn-forming propensity. These results suggest that the discriminative signals in these two tissues are more distributed in nature, and may depend more on the combined balance among residue size, charge, conformational tendency, and polarity/hydrophobicity than on a small number of dominant local dipeptide patterns.

These tissue-specific differences further imply that task separability is determined not simply by which type of feature a model relies upon, but by whether such features form sufficiently stable and distinctive signal structures within a given tissue. Liver provides an illustrative example. It not only showed the best overall predictive performance, but also exhibited a strongly DPC-dominant feature structure, suggesting that Liver-associated ACPs may contain more concentrated and internally consistent local sequence signatures that are easier for the model to learn. By contrast, although Breast and Lung were also highly dependent on DPC features, their overall discriminative performance did not reach the level observed for Liver. This indicates that a DPC-dominant structure does not necessarily imply greater separability. Rather, classification difficulty likely depends not only on whether a model can exploit local motifs, but also on the degree of tissue specificity of those motifs, the internal consistency of samples within a tissue, and the extent of feature overlap with other tissues. Correspondingly, Brain and Prostate, which relied more heavily on integrated physicochemical features, showed relatively weaker predictive performance, suggesting that their tissue-associated signals may be more continuous and diffuse, and less dominated by a small number of highly discriminative local patterns.

From the perspective of features recurring across tissues, the patterns learned by XGBoost do not appear to represent entirely isolated tissue-specific rules, but rather tissue-dependent reconfigurations built upon a shared ACP framework. Among the top 20 features, DPC_IA and DPC_RF each recurred in four tissues, while DPC_LP, DPC_KF, and DPC_LI also appeared repeatedly across multiple tissues. These recurring dipeptides were enriched in hydrophobic or aliphatic residues such as I, L, V, F, and A, as well as positively charged residues such as K and R. This pattern is broadly consistent with the general understanding of ACP biology, namely, that cationicity, hydrophobicity, and their coordinated spatial arrangement are fundamental determinants of membrane interaction and anticancer activity. Tissue specificity, therefore, does not appear to arise from an entirely novel set of rules detached from the canonical physicochemical basis of ACPs; rather, it is more likely to reflect further weighting and refinement of local sequence patterns within a shared membrane-active framework.

Taken together, these observations support the view that differences among tissue-specific ACPs may reside less in whether certain fundamental properties are present, and more in how these properties are locally organized. Classical ACP-related characteristics, including charge, hydrophobicity, aromaticity, residue size, and conformational propensity, do not disappear across tissues; instead, they appear to be recruited in different combinations and local arrangements. Some tissues seem to depend more strongly on local adjacency between positively charged and hydrophobic residues, whereas others rely more on the overall balance of residue size and turn-forming propensity, and still others reflect a joint contribution of sequence-order factors and physicochemical background. This suggests that tissue context may impose constraints on ACP sequences not by replacing the canonical principles of ACP function, but by fine-tuning them within a shared functional framework.

These findings may also offer useful guidance for subsequent sequence optimization and functional design. For tissues such as Liver, Breast, and Lung, where discrimination was clearly driven by DPC features, future work may profitably focus on local motifs, for example, by screening recurrent dipeptide patterns, evaluating the effects of adjacent residue substitutions on model output, or experimentally testing the contribution of local sequence order through mutational analysis. By contrast, for tissues such as Brain and Prostate, where PCP and AAC features contributed more substantially, a more effective strategy may be to optimize overall physicochemical balance, such as residue-size composition, the proportion of positively charged residues, or conformation-related properties, rather than focusing narrowly on a single local motif. In this sense, the design entry points for different tissues may not be identical, which itself reflects the diversity of sequence constraints associated with tissue-specific ACPs.

These interpretations should nevertheless be made with caution. Feature importance reflects the extent to which particular descriptors are utilized by a model during prediction, rather than demonstrating that these features have been directly established as causal determinants at the biological level. This is particularly true for individual DPC features, whose importance should be regarded as a source of hypotheses for subsequent motif analysis and mechanistic validation, rather than as definitive mechanistic evidence. In addition, a degree of redundancy or correlation may exist among different descriptors, and the importance assigned by tree-based models may be influenced by feature collinearity. Figure 5 is therefore best understood as a structured hypothesis-generating framework: it indicates the levels at which tissue-specific signals are most likely to reside and provides a more focused set of candidates for future experimental investigation.

Overall, the feature-importance analysis not only further supports the predictive advantage of XGBoost, but also suggests that XGBoost may be better suited to uncovering the key signal structure underlying tissue-specific ACP discrimination. Compared with RF, which places greater emphasis on aggregated global statistical features, XGBoost appears more capable of extracting discriminative patterns from local sequence organization, and such patterns are likely to constitute an important basis on which tissue-related differences are encoded and recognized. XGBoost is therefore not only the best-performing predictive model in the present study, but also provides a more informative analytical perspective for understanding sequence regularities associated with tissue-related ACP activity.

### 3.4 Cross-tissue analysis

To further examine the balance between shared and tissue-specific determinants, we analyzed the overlap of top-ranked features across the nine tissue-specific XGBoost models. Overall, feature overlap was limited: only a relatively small subset of descriptors recurred across multiple tissues, whereas most high-importance features were retained in only one or a few models. This pattern suggests that, although certain biochemical properties may contribute broadly to anticancer peptide activity, the discriminative signals most relevant for tissue-aware prediction are not uniformly distributed across tissues. Rather than being governed by a single universal sequence rule, tissue-specific ACP activity appears to arise from a shared physicochemical foundation that is differentially weighted and locally reconfigured in distinct tissue contexts (Hoadley et al., 2018).

This point is important because all tissue-specific models were constructed within the same descriptor space and under the same one-versus-rest framework. If broad ACP-associated properties alone were sufficient to explain tissue discrimination, a much greater degree of feature overlap would be expected across tissues. The fact that overlap remained restricted even under these common modeling conditions suggests that tissue specificity is not merely a weak extension of general ACP activity, but instead reflects additional layers of sequence organization superimposed on a conserved anticancer peptide backbone. In this sense, the cross-tissue analysis supports a view of tissue-specific ACPs as partially specialized variants within a broader ACP sequence landscape, rather than as fully discrete and unrelated peptide classes.

This interpretation is further supported by the analysis of peptides observed in more than one tissue category. Such peptides were relatively uncommon within the integrated dataset, indicating that broadly active ACPs represent only a limited portion of the tissue-resolved sequence space. Peptides detected across multiple tissues appeared to show stronger cationic characteristics, including lysine enrichment, consistent with a more general membrane-interactive capacity. However, their limited representation suggests that strong cationicity alone is unlikely to account for tissue-level activity patterns. Instead, while charge-related properties may provide a common mechanistic baseline, activity within a specific tissue context probably depends on a more refined combination of local sequence motifs, residue composition, and physicochemical balance. Broad membrane activity may therefore be necessary for some peptides, but it is unlikely to be sufficient to explain the more selective patterns captured by the tissue-specific models.

Viewed together with the feature-importance analysis, these observations suggest that tissue specificity is more plausibly encoded through differential arrangement and weighting of shared ACP-related properties than through the presence of entirely tissue-exclusive features. Classical ACP-associated characteristics, such as cationicity, hydrophobicity, and conformational propensity, appear to remain relevant across tissues; however, the manner in which these properties are combined, positioned, and prioritized differs from one tissue context to another. This may explain why certain descriptors recur across several tissues while the overall overlap remains modest. What is shared is the general physicochemical logic of ACP function, whereas what differs is the particular sequence-level implementation of that logic in each tissue environment (Chiangjong et al., 2020).

These findings have two broader implications. First, they provide conceptual support for the ACP-Boost framework by showing that tissue-specific modeling is justified at the sequence level. The models do not simply recover generic ACP properties; rather, they appear to capture differences in how those properties are organized across tissue contexts. Second, they suggest that mixed-tissue ACP classifiers should be interpreted with caution. Although such models may detect broad anticancer-associated sequence characteristics, they are less likely to resolve the subtler determinants that distinguish activity across tissues. As a result, tissue-agnostic models may be useful for identifying general ACP-like properties, but they may obscure the finer sequence patterns that are most relevant for tissue-aware prioritization.

At the same time, the present findings also argue against an overly rigid interpretation of tissue specificity. The limited but non-zero overlap across tissues, together with the existence of a small set of multi-tissue peptides, indicates that tissue-associated ACP activity is not fully partitioned into mutually exclusive categories. Instead, the results are more consistent with a continuum-like structure in which some peptides occupy relatively specialized regions of ACP sequence space, whereas others retain broader cross-tissue potential. From this perspective, the proposed framework is best viewed not as a definitive classifier of fixed biological classes, but as a comparative model for positioning peptides along a tissue-related gradient of sequence preference and prioritizing candidates for further validation.

Several limitations should nevertheless be acknowledged. Under the current one-versus-rest design, peptides associated with other tissues were treated as the reference class rather than general non-ACP peptides. Accordingly, the framework is more appropriately interpreted as a tissue-aware discriminator within ACP-related sequence space than as a standalone screening model for arbitrary peptide libraries. In addition, predictive performance remained moderate for several tissues, particularly those with greater biological heterogeneity or more limited data representation, indicating that tissue-specific signals are only partially separable using sequence-derived descriptors alone. Future studies should therefore expand experimentally validated peptide datasets, incorporate subtype-resolved annotations, and integrate structural, biophysical, and microenvironment-related variables to improve both robustness and biological interpretability. Experimental validation of high-confidence predictions will also be essential for establishing the translational relevance of the present framework.

## 4 Conclusion

In this study, we developed and evaluated a tissue-aware machine learning framework for anticancer peptide (ACP) classification using a one-versus-rest strategy across nine tissue contexts. The results indicate that ACP sequences contain detectable tissue-associated signals, although the degree of separability varies across tissues.

Among the five evaluated classifiers, XGBoost achieved the best overall performance and was therefore selected as the primary model for downstream analysis. Feature-importance analysis further showed that tissue-specific prediction was supported by partially overlapping yet distinct descriptor profiles, suggesting that ACP activity is shaped by both shared physicochemical properties and tissue-dependent sequence patterns.

These findings support the value of incorporating tissue context into computational ACP modeling. At the same time, the current framework should be interpreted as a tissue-aware comparative model within ACP-related sequence space, and its performance remains constrained by data size, class imbalance, and biological heterogeneity. Future studies integrating larger experimentally validated datasets and additional structural or biophysical information may further improve predictive robustness and interpretability.

Overall, this work provides a systematic bioinformatics framework for tissue-specific ACP analysis and highlights the importance of tissue-aware modeling in computational peptide discovery.

## Data Availability

Publicly available datasets were analyzed in this study. The original peptide records were obtained from the CancerPPD and DCTPep databases. The processed data supporting the findings of this study are included in the article/Supplementary Material. Further inquiries can be directed to the corresponding author.

## Supplementary material

SUPPLEMENTARY TABLE S1 Detailed benchmark results of five machine learning algorithms across nine tissue-specific one-versus-rest ACP prediction tasks.

SUPPLEMENTARY TABLE S2 Distribution of descriptor categories among the top 20 features pooled across nine tissue-specific one-versus-rest ACP classification tasks in Random Forest and XGBoost models.

SUPPLEMENTARY FIGURE S1 Top 20 sequence-derived features contributing to tissue-specific ACP classification in Random Forest and XGBoost models across nine one-versus-rest tasks. For each tissue-model combination, features are ranked by importance score, and only the top 20 descriptors are displayed.
