# Supplementary figures and images for: Comparative analysis of tissue-specific anticancer peptide prediction models: ACP-Boost framework
Source: Front Mol Biosci. 2026 Mar 26;13:1815309. doi: 10.3389/fmolb.2026.1815309 (PMC13062271; doi:10.3389/fmolb.2026.1815309)

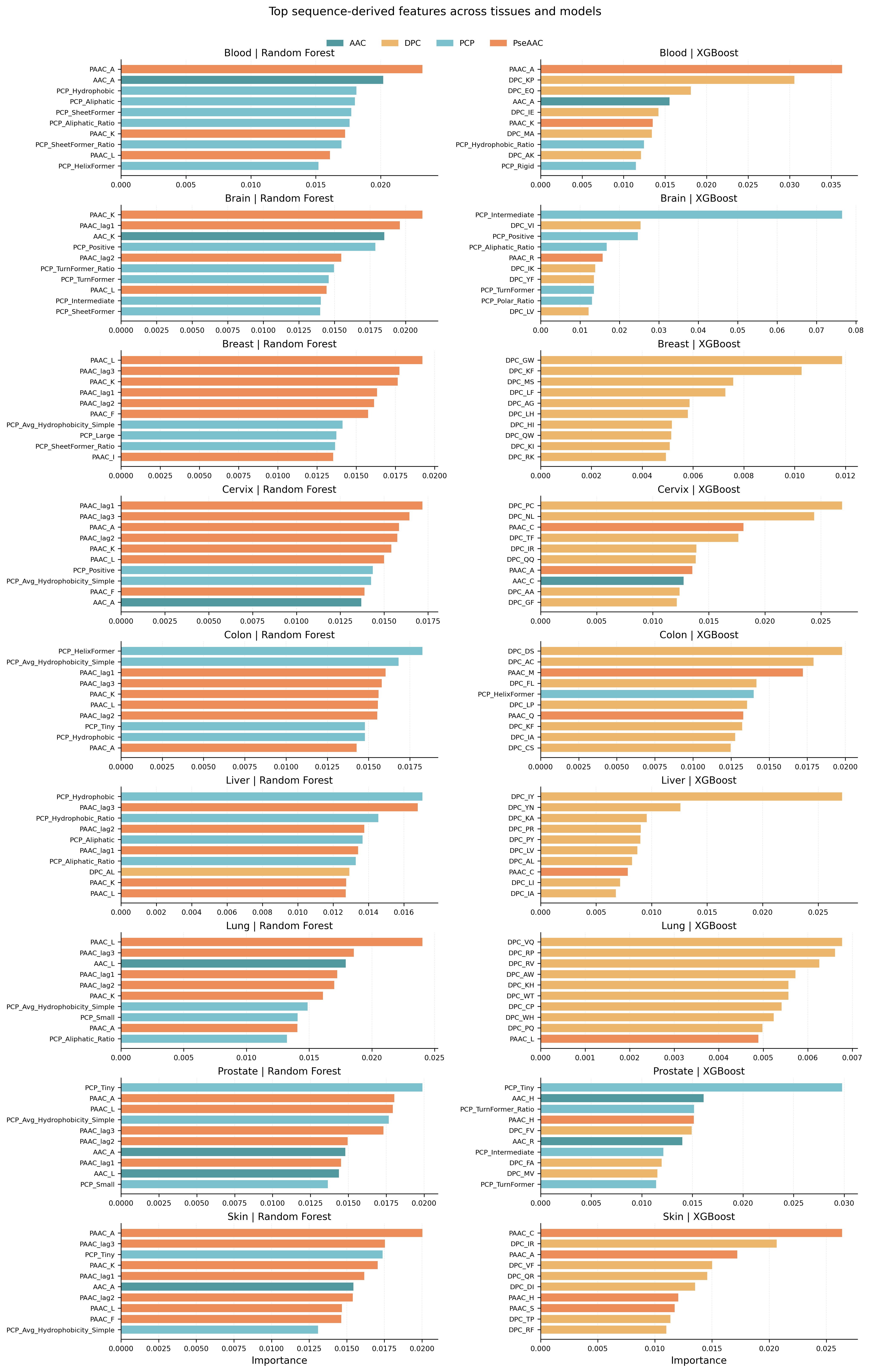

Supplement: Supplementary file 3 [file Image1.png]
